# *ARHGDIA* Confers Selective Advantage to Dissociated Human Pluripotent Stem Cells

**DOI:** 10.1089/scd.2021.0079

**Published:** 2021-07-16

**Authors:** Marion J. Riggs, Steven D. Sheridan, Raj R. Rao

**Affiliations:** ^1^ Department of Chemical and Life Science Engineering, Virginia Commonwealth University, Richmond, Virginia, USA.; ^2^ Center for Genomic Medicine, Massachusetts General Hospital, Boston, Massachusetts, USA.; ^3^ Department of Neurology, Harvard Medical School, Boston, Massachusetts, USA.; ^4^ Center for Quantitative Health, Center for Genomic Medicine and Department of Psychiatry, Massachusetts General Hospital, Boston, Massachusetts, USA.; ^5^ Department of Psychiatry, Harvard Medical School, Boston, Massachusetts, USA.; ^6^ Department of Biomedical Engineering, College of Engineering, University of Arkansas, Fayetteville, Arkansas, USA.

**Keywords:** pluripotent, stem cells, genome instability, self renewal, ARHGDIA, karyotype

## Abstract

Human pluripotent stem cells (hPSCs) have generated significant interest in the scientific community based on their potential applications in regenerative medicine. However, numerous research groups have reported a propensity for genomic alterations during hPSC culture that poses concerns for basic research and clinical applications. Work from our laboratory and others has demonstrated that amplification of chromosomal regions is correlated with increased gene expression. To date, the phenotypic association of common genomic alterations remains unclear and is a cause for concern during clinical use. In this study, we focus on trisomy 17 and a list of candidate genes with increased gene expression to hypothesize that overexpressing 17q25 located *ARHGDIA* will confer selective advantage to hPSCs. HPSC lines overexpressing *ARHGDIA* exhibited culture dominance in co-cultures of overexpression lines with nonoverexpression lines. Furthermore, during low-density seeding, we demonstrate increased clonality of our ARHGDIA lines against matched controls. A striking observation is that we could reduce this selective advantage by varying the hPSC culture conditions with the addition of ROCK inhibitor (ROCKi). This work is unique in (1) demonstrating a novel gene that confers selective advantage to hPSCs when overexpressed and may help explain a common trisomy dominance, (2) providing a selection model for studying culture conditions that reduce the appearance of genomically altered hPSCs, and (3) aiding in elucidation of a mechanism that may act as a molecular switch during culture adaptation.

## Introduction

Human pluripotent stem cells (hPSCs) are defined by their ability to self-renew, undergo tri-lineage differentiation, and maintain a normal karyotype [1]. However, earlier results from our laboratory and others demonstrate a propensity for hPSCs to acquire abnormal genomic signatures upon prolonged propagation [2–5]. Moreover, the presence of genomic alterations can be increased by specific hPSC passaging methodologies [4,6]. Two general approaches for passaging hPSCs involve enzymatic dissociation into single cells or separation into small clumps. The poor survival of hPSCs under single-cell dissociation is well documented [7,8].

Passaging hPSCs as single cells has the following advantages: (1) increased numbers for scale-up, (2) standardization of differentiation protocols, and (3) clonal genetic manipulation [8–11]. Unfortunately, increased genomic instability during single-cell passaging is a primary concern and poor single-cell viability adversely affects protocols and impacts large-scale production [4,12]. To increase hPSC single-cell survival at passaging, many researchers have incorporated small molecules in the culture medium based on inhibiting RHOA-ROCK-pMLC pathway to improve single-cell plating efficiencies and reduce apoptosis [13]. However, use of ROCK inhibitor (ROCKi) in culture medium is contentious, since apoptosis is proposed to purge cultures of cells with genomic damage and chromosomal alterations [14–16].

Strong selective pressure may exist for genomic alterations that increase clonal survival and are antiapoptotic [17,18]. Indeed, the high recurrence of specific genomic species in hPSC culture, such as trisomy 12, 17, and 20, is consistent with strong selective pressure suggesting that in hPSC culture adaptation, comprising of mutation followed by selection, selection is a particularly strong force in the emergence of genomic variants [3]. To date, the phenotype behind common genomic alterations has been unclear, and more specifically, the functionally relevant genes located on these genomic loci causative for culture selection have remained elusive [4].

We take the approach of passaging hPSCs as single cells to (1) reproducibly generate genomic abnormalities for further study, (2) better understand single-cell passaging's influence on genomic instability, and (3) determine conditions that may reduce genomic instability during single-cell passaging. We hypothesize that increasing cell viability will reduce selection of genomic variants and promote propagation of genomically normal hPSCs. This work is unique in demonstrating that increasing expression of *ARHGDIA*, a gene located on chromosome 17q25, confers selective advantage to hPSCs, and by providing a culture selection model for studying conditions potentially reducing the appearance of genomically altered hPSCs.

## Materials and Methods

### Cell culture, stem cell characterization, and karyotype analysis

HPSC lines BG01, H1, H9, iPSC (IMR-90) (WiCell Research Institute, Madison, WI), and BG01(v) were maintained on inactivated mouse embryonic fibroblasts (iMEFs) or BD Matrigel in DMEM/F-12, 20% knockout serum replacement, 2 mM l-glutamine, 1% nonessential amino acids, 50 U/mL penicillin, 50 μg/mL streptomycin (all from Gibco/Invitrogen), 0.1 mM beta mercaptoethanol (Sigma), and 4 ng/mL bFGF (Sigma). For BD Matrigel, the medium was conditioned on iMEFs. Cells were enzymatically passaged by sequential dissociation using 1 mg/mL type IV collagenase (Gibco) and 0.05% trypsin-ethylene-diamine tetra-acetic acid (trypsin-EDTA; Invitrogen) or manually passaged by fire-pulled Pasteur pipette.

G-banding, stem cell characterization, and embryoid body (EB) differentiation protocols are previously described [19] and further details can be found in Supplementary Data S1. Before the single-cell dissociation and genomic instability experiments, normal diploid karyotype for BG01, H1, H9, and iPSC (IMR-90) was directly assessed in this study or previously reported by our laboratory as follows: BG01 [3], H1 (Supplementary Table S1), H9 [19], and iPSC (IMR-90) [19]. In accordance with federal regulations regarding the protection of human research subjects (32 CFR 219.101(b)(4)), and due to the fact that the cell lines used were from sources part of the NIH stem cell registry, the VCU Office of Research Compliance determined that the project was exempt from Institutional Review Board (IRB) oversight and human research subjects protection regulations.

### Microarray analysis

Statistical analysis was performed in the R environment. Arrays were normalized by cyclic loess and signal intensities summarized by GCRMA. *P* values were adjusted by Benjamini and Hochberg correction with significance determined at an FDR <0.05. See Supplementary Data S1 for further detail on the arrays, chromosomal distribution, and ontology analysis.

### Generation of ARHGDIA overexpression lines

LentiORF *ARHGDIA* w/Stop Codon (Open Biosystems) was used for the expression construct. The plasmid was purified using Qiagen Maxi Prep. Lentivirus was generated using HEK293 cells with psPAX2 and pMD.2 plasmids. The viral supernatant was concentrated using the Lenti-X concentrator. Lentivirus was added to hPSCs in the presence of polybrene. See Supplementary Data S1 for further detail.

### Competition assay

HPSCs (Arg) and hPSC (WT) were maintained independently by manual passaging until use in specific experiments. Upon initial enzymatic passage, cells were 40 μm filtered and seeded as mixed cultures onto Matrigel™ or iMEFs. At each passage, cultures were enzymatically dissociated into single cells, 40 μm filtered, and the hPSC (Arg) percentage quantitated by flow cytometry using the Accuri C6 instrumentation. The percentage of GFP-positive hPSCs was gated against control plots of hPSC (WT) cells. See Supplementary Data S1 for further detail.

## Results

### Genomic alterations are observed under single-cell passaging

**FIG. 1.**
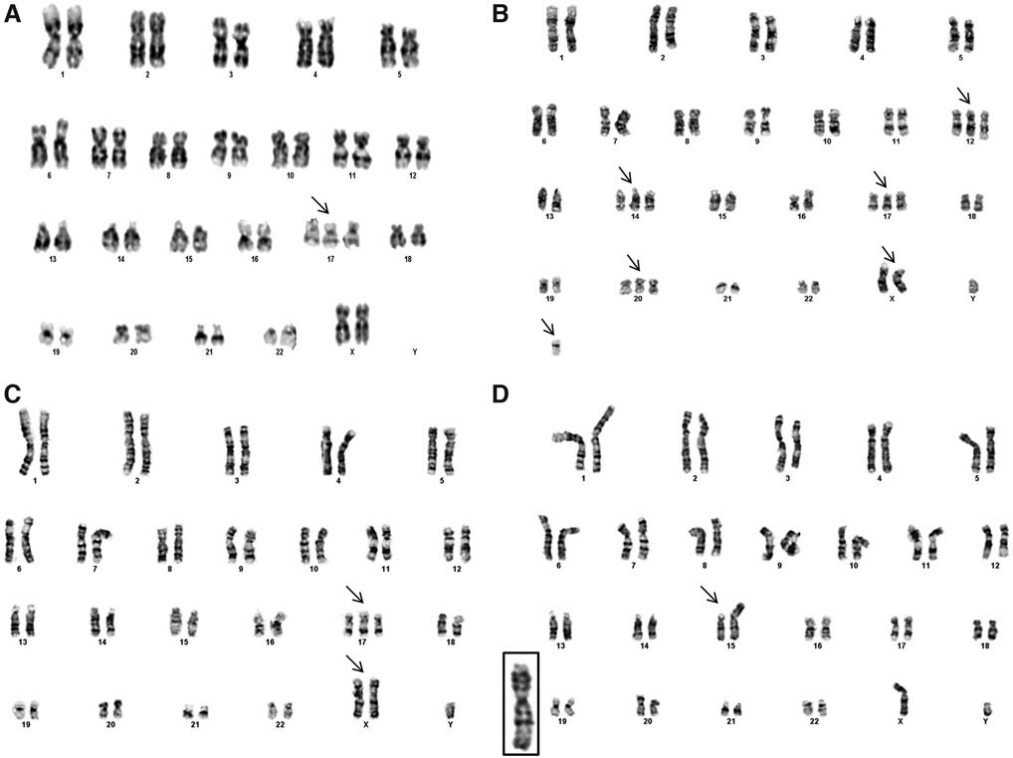
Karyogram of four hPSC lines serially passaged as single cells. H9 line at passage 71 corresponding to E-26 exhibiting trisomy 17 **(A)**. BG01(v) line exhibiting multiple trisomies on 12, 14, 17, 20, and X and a +der(16) **(B)**. hiPSC exhibiting trisomy 17 and gain of X **(C)**. H1 line with a segmental duplication on 15p11.2 **(D)**. hPSC, human pluripotent stem cell.

For all experiments conducted, g-banding confirmed a normal karyotype for hPSC lines: BG01, H1, H9, and iPSC (IMR-90), as shown by our laboratory [3,19]. Euploid hPSC lines were manually dissociated as aggregates for replating before initiating our genomic instability experiments. For genomic instability studies each line, H1, H9, iPSC (IMR-90), and BG01(v), was enzymatically (E) dissociated into single cells and seeded onto iMEFs. Prior observations from our laboratory suggest 25 passages are sufficient to observe genomic abnormalities [3], so we initiated g-band karyotyping after this enzymatic passage window (Fig. 1 and Supplementary Table S1).

Karyotyping the BG01(v) line validated the existence of previously reported genomic alterations on chromosomes 12, 14, 17, and X. Under continuous culture, the H9 line was karyotyped at two passages, E-26 and E-105. At E-26, 67% of the karyotyped H9 cell line exhibited trisomy 17, and at E-105, trisomy 17 and 12 were identified, suggesting an accumulation of genomic alterations upon extended culture. The H9 associated trisomy 17 at passage E-105, contained a deletion of p11.2. Trisomy 17 mosaicism of the H9 line at E-26 suggests capture of the initial culture dominance of this genomic species. The iPSC line, at passage E-31, was trisomy for 17 and harbored a gain of X. The H1 line was karyotyped at two enzymatic passage points, E-29 and E-58. While the initial H1, E-29 sample was euploid, at E-58, we found a previously unreported structural gain on chromosome 15 at p11.2.

### Candidate gene identification for positive selection of trisomy 17

Culture dominance by variant hPSCs arises from phenotypic selection with genotypic association [17]. Therefore, we performed transcriptome analysis using Affymetrix HGU-133a microarrays to identify transcriptional patterns and candidate genes conferring selective advantage. Differential gene analysis of the trisomy BG01(v) line against the previously reported euploid H1 line [20,21] demonstrated an enrichment of significantly increased genes on the amplified chromosomes compared to the diploid chromosomes, *P* value = 0.0046, confirming that transcription levels correlate with genomic copy number (Supplementary Fig. S1).

We further sought to determine potential biomarkers. Utilizing four databases Gene Ontology [22], PluriNet [23], Cancer Genome Project [24], and Genomic Instability [25] for a priori information, we collected a list of annotated genes to intersect with our differential gene expression analysis. Approximately 2,031 genes were included, in which we focused on those genes with an FDR <0.05, located on autosomal chromosomes 12, 14, or 17 (Table 1).

**Table 1.** Significantly Increased Genes Located on Trisomy Chromosomes in BG01(v) Line

Symbol	Cytoband	Ontology	Phenotype
KRAS	12p12.1	Ras-mediated signal transduction	Cancer
SOCS2	12q	**Antiapoptosis**	
DYRK2	12q15	**DNA damage response, induction of apoptosis**	
RFC5	12q24.2-q24.3	**DNA repair, DNA replication, checkpoint sensor**	Genomic instability, self-renewal
PSMD9	12q24.31-q24.32	**Mitotic cell cycle**	
POLE2	14q21-q22	**DNA repair**	
GPHN	14q23.3	establishment of synaptic specificity at neuromuscular junction	Cancer
ALKBH1	14q24.3	**DNA dealkylation**	
DYNC1H1	14q32.3-qter	**Mitotic spindle organization and biogenesis**	
USP22	17p11.2	**Positive regulation of mitotic cell cycle**	
RNMTL1	17p13.3	RNA processing	Self-renewal
PAFAH1B1	17p13.3	**Establishment of mitotic spindle orientation**	
RPA1	17p13.3	**DNA repair, checkpoint signaling**	Cancer predisposition, genomic instability, self-renewal
NF1	17q11.2	**Positive regulation apoptosis, negative regulation of cell proliferation**	Cancer
TIAF1	17q11.2	**Antiapoptosis**	
PSMD11	17q11.2	**Mitotic cell cycle**	Self-renewal
COL1A1	17q21.33	extracellular matrix	Cancer
RAD51C	17q22-q23	**DNA repair**, **HR**	Genomic instability
SOX9	17q24.3-q25.1	**Apoptosis, Cell proliferation**	
SEP9	17q25	Cell cycle, GCPR signal transduction	Cancer
EXOC7	17q25.1	**Centriolar satellite**	
ARHGDIA	17q25.3	**Antiapoptosis**	

Significant genes located on chromosomes 12, 14, or 17 with an FDR <0.05. A gene's ontology is bold if part of genomic instability [22] or an annotation of interest from Gene Ontology. Self- renewal genes are from the PluriNet gene set and phenotype cancer from the Cancer Genome Project.

HR, homologous recombination.

Chromosome 17 is one of the most common genomic abnormalities in hPSCs, occurring in vitro during hPSC culture and *in vivo* in human embryonic carcinoma cells [26]. In our experiments, three hPSC lines [BG01(v), H9, and iPSC] exhibited trisomy 17 during long-term propagation. In addition, published reports suggest that 17q25 may be a minimal amplicon for genomically altered hPSCs [17]. Interestingly, 17q25 is reported to be the only species conserved genomic amplification between *homo sapiens* and syntenic locus of *mus musculus* and *rhesus macaque* [27]. Therefore, we focused on the significant genes located on 17q25.

Of the three overexpressed genes, SEPTIN 9, EXO7, and *ARHGDIA*, *ARHGDIA* caught our attention for its established role in the RHO-ROCK pathway [12,13,28]. ROCKi is commonly used to reduce dissociation-induced cell death resulting from loss of e-cadherin-mediated cell-cell contact [8]. ARHGDIA inhibits the activation of RHOA by preventing the GDP exchange for GTP [29]. Since RHOA activation is necessary for ROCK activation, we hypothesized that overexpression of *ARHGDIA* would reduce activation of RHOA and therefore lead to increased single-cell survival conferring selective advantage to hPSCs (Supplementary Fig. S2).

### ARHGDIA-transduced cell lines maintain pluripotency

To test our selective advantage hypothesis, we generated, by lentiviral transduction, two hPSC lines, H9 (Arg) and BG01 (Arg), which constitutively overexpress *ARHGDIA*, and similarly generated matched controls for GFP reporter only, H9 (GFP) and BG01 (GFP). Both hPSC (Arg) lines demonstrate increased ARHGDIA expression by quantitative polymerase chain reaction (qPCR) and Western blot, validating our experimental system (Supplementary Figs. S8 and S9). By densitometry, fold increase on ARHGDIA protein abundance was 3.74 for BG01 (Arg) and 10.52 for H9 (Arg). In the variant lines, the comparative increase in ARHGDIA levels for the BG01(v) and H9 (v) lines is 2.68 and 2.12, respectively.

**FIG. 2.**
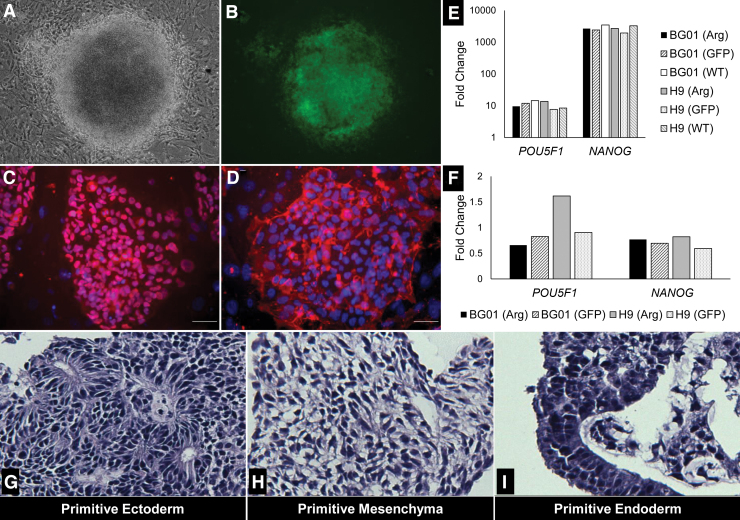
HPSCs overexpressing *ARHGDIA* demonstrate hallmark pluripotency. H9 (Arg) exhibits dense and tightly packed standard pluripotent stem cell colony formation **(A)** and concomitant GFP signal indicating positive *ARHGDIA* overexpression **(B)**; magnification is 4 × . Positive nuclear expression of OCT4 and cell surface marker SSEA4, **(C, D)**, respectively; DAPI-*blue* **(C, D)**, OCT4-*red* **(C)**, SSEA4-*red* **(D)**; Scale bar = 50 μm. HPSC transcription factor gene expression relative to human dermal fibroblast **(E)**. hPSC (Arg) experimental and HPSC (GFP) controls were validated for hPSC transcription factor gene expression against WT lines **(F)**. For each cell line, *n* = 3 **(E, F)**. Hematoxylin and eosin-stained histologic sections of EBs from H9 (Arg) lines. The trilineage differentiation is indicative of pluripotency **(G**–**I)**. Magnification is 20 × . Arg, ARHGDIA; EBs, embryoid bodies; GFP, green fluorescent protein; WT, wild type. Color images available.

To confirm that *ARHGDIA* overexpression did not adversely influence self-renewal and differentiation, we characterized our hPSC (Arg) lines for pluripotency. Our transduced lines exhibit typical hPSC cobblestone, colony morphology on iMEFs (Fig. 2A, B) and Matrigel (data not shown). Interestingly, the H9 (Arg) colonies on iMEFs appeared to have increased cell-cell contact, demonstrated by greater multilayer colony density (Supplementary Fig. S3). After 30 passages, the hPSC (Arg) lines exhibited by immunocytochemistry positive protein expression for characteristic stem cell markers, OCT4 and SSEA4 (Fig. 2C, D and Supplementary Figs. S4 and S5). When comparing gene expression by qPCR of *OCT4* and *NANOG* in hPSC (Arg) lines against human dermal fibroblasts (hDFs), we observed fold changes of one and two orders of magnitude, respectively. Across hPSC lines, the fold change patterns against the hDFs were similar. Quantitative PCR of *NANOG* and the OCT4 gene *POU5F1* in H9 (Arg) and BG01 (Arg) relative to hPSC (GFP), hPSC (WT), and hDF controls confirms self-renewal of these lines (Fig. 2E, F). Through EB formation, we validated by histopathology that H9 (Arg) EBs stained for all three primitive germ layers, indicating hPSC (Arg) lines retained their differentiation capacity (Fig. 2G–I). Taken together, these results indicate *ARHGDIA* overexpression does not adversely affect pluripotency.

### ARHGDIA confers selective advantage and increases clonality

For the H9 (Arg) line, we used two assays to test our hypothesis that *ARHGDIA* overexpression will improve hPSCs cultured as single cells: the first, a competition-based assay, consisted of co-cultures of cells overexpressing *ARHGDIA* versus nonoverexpressing cells in which the transduced *ARHGDIA* cells co-express GFP. The second assay tested clonality of single cells seeded at low density.

Competition co-culture experiments were performed on two independent H9 (Arg) sublines, H9 (Arg) s.1 and H9 (Arg) s.2. For each subline, the competition-based assay was initiated as a mixture of H9 [Arg(+)] and H9 [Arg(−)] co-cultures, discriminated by GFP co-expression. At each passage, the percentage of GFP-positive cells was measured by flow cytometry. Both H9 (Arg) s.1 and H9 (Arg) s.2 sublines exhibited strong competitive advantage when passaged as single cells. The initial subpopulations of H9 (Arg+) s.1 and H9 (Arg+) s.2 were 53.2% and 42.0%, respectively. After serial, single-cell passaging, the total H9 [Arg(+)] percentages for the H9 (Arg) s.1 and H9 (Arg) s.2 lines reached 90.6% and 93.5%, respectively (Supplementary Figs. S6 and S7).

**FIG. 3.**
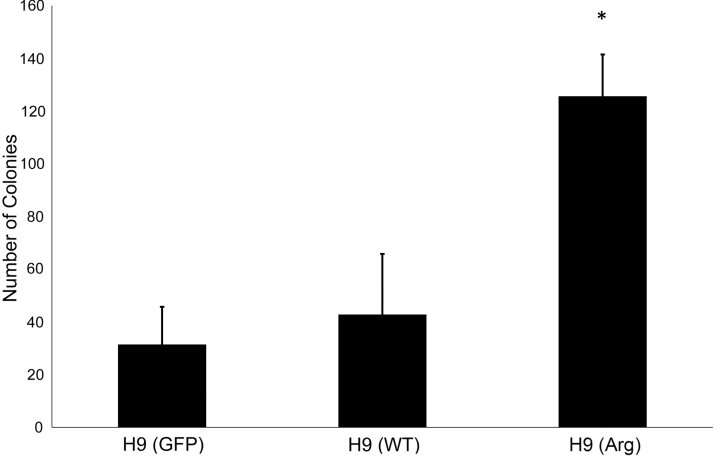
*ARHGDIA* overexpression increases clonality. H9 control and experimental lines were continually propagated by manual dissection before clonal survival analysis. H9s were plated at 100 cells/cm^2^ under initial enzymatic dissociation and 40 μm filtering. Colonies were AP stained and counted on day 7. H9 (Arg) line overexpressing *ARHGDIA* has significantly increased number of colonies in low-density single-cell seeding. *Indicates significance (*P* < 0.01) for H9 (Arg) compared to H9 (WT) and H9 (GFP): *N* = 10, *N* = 8, and *N* = 9, respectively. AP, alkaline phosphatase.

Next, we determined whether H9 (Arg) cells exhibited increased clonality. H9 (Arg), H9 (GFP), and H9 (WT) were manually passaged until clonal analysis. Clonality was tested on the first enzymatic passage to control for selective pressure. On day 7, colonies were alkaline phosphatase (AP) stained to aid visualization and validate pluripotency [30]. The observed average colony numbers for H9 (Arg), H9 (GFP), and H9 (WT) are 125.66, 31.44, and 42.83, respectively (Fig. 3). Therefore, clonality of H9 (Arg) cells increased 4.0- and 2.93-fold relative to H9 (GFP) and H9 (WT) with *P* values of 0.0023 and 0.0004, respectively. Together, the competition and clonality experiments support the conclusion that H9 (Arg) cells have competitive advantage over H9 (WT) cells.

We repeated our competition-based assay on BG01 (Arg) cells to test variable culture conditions, including substrates (i-MEFS and BD Matrigel, seeding density (25K, 50K, and 100K per 35 mm), and ROCKi exposure. Competitive advantage of BG01 (Arg) cells in mixed co-cultures with BG01 (WT) is strikingly observed. In contrast, co-culture experiments of BG01 (GFP) against BG01 (WT) did not exhibit competitive advantage. Each co-culture experimental condition was carried out in parallel biological triplicates.

**FIG. 4.**
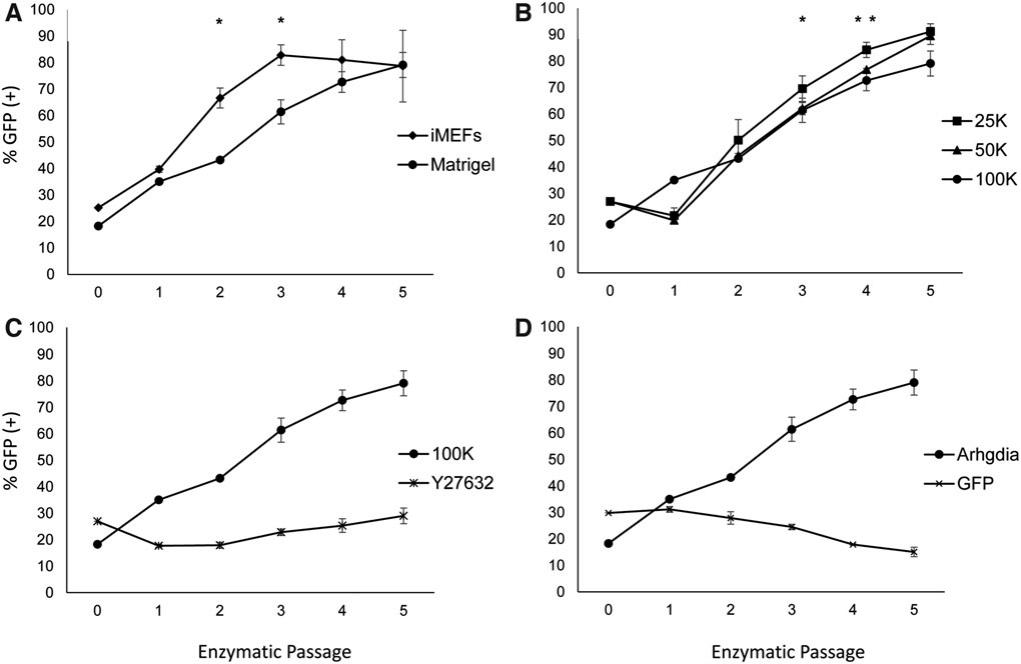
BG01 (Arg) hESCs demonstrate competitive advantage across substrates and seeding densities and selection is inhibited by ROCKi, Y27632. BG01 (Arg) lines co-cultured with BG01 (WT) have competitive advantage across substrates **(A)** and seeding densities in 35 mm Petri dishes **(B)**. ROCKi, ameliorated competitive advantage **(C)** and BG01 (GFP) control did not exhibit competitive advantage **(D)**. ROCKi, ROCK inhibitor.

In their feeder-free cultures, Rosler and colleagues report trisomy 20 as the most frequently observed aberration [31], while trisomy 17 is prominent in our hPSC cultures propagated on iMEFs. Thus, we sought to determine whether iMEFs or Matrigel influenced competitive advantage of BG01 (Arg) versus BG01 (WT) co-cultures. Both substrates showed strong selection for BG01 (Arg) cells. In serial seedings at 100K, by E-3, the BG01 (Arg) cells dominated to 82.87% on iMEFs and comparatively on Matrigel increased to 79.11% at E-5. For both E-2 and E-3, the percent BG01 (Arg) is significantly higher in the iMEF cultures compared to Matrigel, *P* < 0.01 (Fig. 4A). These results suggest more broadly that substrates can influence culture selection of hPSC genomic variants.

Next, we investigated seeding density on BG01 (Arg) and BG01 (WT) co-cultures plated at 25K, 50K, and 100K per 35 mm on Matrigel. Since, low single-cell seeding decreases survival of hPSCs possibly by reduced paracrine signaling [32] or increased cell migration distance [33,34], relative competitive advantage of the BG01 (Arg) to the BG01 (WT) may be greater in co-cultures as seeding density decreases. BG01 (Arg) competitive advantage was clearly demonstrated within five enzymatic passages for each seeding density. The maximal BG01 (Arg) percentages for 25K, 50K, and 100K, are 91.19%, 89.52%, and 79.11% (Fig. 4B). The higher percent of BG01 (Arg) in the 25K samples relative to 50K is significant for E-4 (*P* < 0.05) and is significant relative to 100K for E-4 and E-5 (*P* < 0.05). This supports the existence of subtle selection pressure at lower seeding densities.

Finally, we tested the impact of ROCKi, given its role in promoting single-cell viability during plating. We hypothesized that ROCKi would reduce the selective advantage of BG01 (Arg) cells relative to BG01 (WT) by increasing the survival of dissociated BG01 (WT) cells. BG01 (Arg) and BG01 (WT) co-cultures were seeded at 100K on Matrigel in the presence of 10 μM Y27632 containing growth medium. Strikingly, when ROCKi was added to the cultures during plating and subsequently withdrawn at the first medium exchange, we did not observe competitive advantage of BG01 (Arg) cells (Fig. 4C). Starting with an initial BG01 (Arg) percentage of 26.97%, by E-5, BG01 (Arg) cells remained similar at 29.02% of culture. This is in stark contrast to our other BG01 (Arg) experiments, in which competitive advantage was demonstrative. We checked whether BG01 (GFP) control line subpopulations displayed competitive advantage and indeed this was not the case (Fig. 4D).

Combined across BG01 (Arg) and H9 (Arg) co-culture and clonality experiments, our results present compelling evidence that *ARHGDIA* overexpression confers selective advantage to hPSCs through increased single-cell survival, and this selective advantage can be ameliorated by the addition of ROCKi.

## Discussion

Trisomy 17 is the most common abnormality observed in our hPSC lines and was scored in the H9, BG01v, and iPSC lines. The recurrent trisomy 12 was also observed in the BG01(v) and H9 lines. *NANOG* is located on chromosome 12 and increased *NANOG* expression may confer self-renewal benefit to these populations [35]. Of note, trisomies 12 and 17 are hallmarks of germ cell tumors and the presence of these alterations in hPSCs as well as other known variations poses potential clinical concern [26,36,37]. Enzymatic passage may increase genomic instability more than twofold [4]. We observe disproportionate expression of trisomy 17 under single-cell dissociation potentially indicating that a chromosome 17 gain is associated with a clonal phenotype.

Significantly increased genes on recurrent genomic amplifications are candidates for biological relevance in positive selection. In our analysis, *ARHGDIA* was the most attractive gene for its location, gene expression, and established role in RHOA signaling [12]. Consistent with trisomy, our study is the first to demonstrate that increased expression of a gene located on chromosome 17 confers selective advantage to hPSCs when passaged as single cells.

*ARHGDIA* overexpression did not seem to adversely affect self-renewal or differentiation, as *ARHGDIA*-transduced cultures were maintained for several months and differentiated into EBs comprising all three germ layers. This is consistent with the routine use of ROCKi for hPSC culture; however, some laboratories have begun to report using ROCKi in differentiation protocols [38–41].

In H9 cell lines, we tested two independent approaches for ARHGDIA's influence on survival. The H9 [Arg(+)] cells demonstrated competitive advantage against H9 [Arg(−)] cells. In the H9 clonality assay, single-cell survival was drastically increased in H9 (Arg) cells compared to controls strongly supporting clonality as the phenotypic advantage.

The BG01 (Arg) cells dominated co-cultures in as quickly as three passages in the iMEF condition. Across seeding densities on Matrigel, the BG01 (Arg) cells with the lowest seeding density of 2.5 k/cm^2^ exhibited the fastest culture dominance. Both hPSC migration promoting cell-cell contact [33] and increased trophic paracrine signaling are thought to increase hPSC viability [32,42]. Either of these factors may have reduced the survival of BG01 (WT) at lower seeding densities with a relative increase in selective advantage of the BG01 (Arg) cells. This poses interesting questions regarding the mechanism in which increased levels of ARHGDIA improves survival during single-cell plating, such as through possibly inhibiting ROCK-associated blebbing, facilitating migration, or in an unidentified cell adhesion factor affecting clonal survival.

Ben-David et al. suggest that ROCKi may reduce the rate of genomic adaptation of hPSCs in culture by reducing selective pressure [43]. However, to date there has not been any report directly testing this hypothesis. Of note, Thompson and colleagues did not observe an increase in hPSC mutation frequency when culturing with Y27632 [16]. Our experiments are the first to provide compelling evidence that ROCKis can be used to reduce the selection of competitive subpopulations.

Interestingly, through live-cell imaging, Barbaric and colleagues elucidate the impact of culture adaptation on alleviating the bottlenecks of colony formation with implications for selective advantage of variant hPSCs [44]. Consistent with the observations in our ROCKi co-culture experiments, Y27632 had negligible prosurvival influence on their aneuploid H7 and H14 cultures. Noteworthy, the H7 and H14 lines used in their study are known for trisomy 17, spanning the region containing *ARHGDIA*. Thus, our two studies may be uncovering similar mechanisms for culture adaptation that we propose is through the RHOA-ROCK-pMLC pathway. While our study emphasizes single-cell survival, it will be of keen interest to investigate, in further detail, ARHGDIA's potential influence on adhesion, migration, and cell cycle kinetics.

The variability of genomic species and time to emergence poses inherent challenges to statistically assess culture conditions influencing hPSC culture genomic adaptation. Several reports have shown that *BCL-XL* increases the survival of hPSCs and mediates the selective advantage of 20q11.21 amplicon by increasing expression levels [32,45,46]. Together, *ARHGDIA* and *BCL-XL* provide biologically relevant candidates for engineering hPSC lines to study culture conditions influencing positive selection of genomic variants. Such genetically defined lines can be used to reproducibly test small molecules, substrates, and three-dimensional culture systems on amenable time scale.

HPSCs are known for high rates of centrosomal instability [47] and genomically mosaic cultures [48,49] indicating an inherent and appreciable mutational background. Thus, for prolonged propagation of genetically normal hPSCs, improving culture conditions to reduce selective advantage of variants is a particularly attractive strategy.

## Conclusions

We observe common trisomy 17 in our hPSC cultures and increased gene expression associated with amplified chromosomes. We demonstrate that increasing gene expression of 17q25-located *ARHGDIA* confers strong selective advantage to hPSCs under enzymatic passage plated as single cells. Our hPSC (Arg) cells exhibit clear increased single-cell survival in our clonality assay and reproducibly dominate our mixed co-cultures. Overexpression of *ARHGDIA* does not affect pluripotency. We show that subtle culture conditions influence selective advantage of our genetically modified line. Using Y27632 to improve survival of hPSCs ameliorates clonal disadvantage of wild-type hPSCs and reduces selective advantage of our ARHGDIA hPSC lines.

## Supplementary Material

Supplemental data

Supplemental data

Supplemental data

Supplemental data

Supplemental data

Supplemental data

Supplemental data

Supplemental data

Supplemental data

Supplemental data

Supplemental data
