# Supplementary material for: ARHGDIA Confers Selective Advantage to Dissociated Human Pluripotent Stem Cells
Source: Stem Cells Dev. 2021 Jul 16;30(14):705–13. doi: 10.1089/scd.2021.0079 (PMC8309423; doi:10.1089/scd.2021.0079)
Supplement: Supplemental data [file Supp_Data1.docx]

SUPPLEMENTAL METHODS

**Microarray Gene Expression Analysis**

Statistical analysis was performed in the R environment. 6 HGU- 133A Affymetrix microarrays comprising two classes, BG01v’s (aneuploid) and H9’s (euploid), were normalized by cyclic loess and signal intensities summarized by GCRMA. Presence (P), marginal (M), and absence (A) calls were determined using the MAS 5.0 detection algorithm. A gene was considered for hypothesis testing, if it was considered present in either sample class. A gene was considered present in either the normal or abnormal set if it was called present in at least two of the three replicates. P-values were adjusted via Benjamini and Hochberg correction. Statistically significant genes were determined at a FDR less than 0.05. Fold changes were calculated in reference to the euploid sample arrays, such that positive fold change values reflect an increase in expression in the aneuploid class and negative fold changes an increase in the relative expression of the normal class.

**Chromosomal Distribution and Fold Change Calculation**

Each probe set was annotated with chromosomal location via R using the *hgu133a.db* library. A normalized ratio for each chromosome was plotted on a histogram, with the values calculated by dividing the number of significant genes (either increased or decreased) by the number of known probe sets for each respective chromosome. Probe sets considered present among the normal class were considered for chromosomal fold change analysis. For each chromosome, the mean across replicates was found for each gene, then the 0.2 trimmed mean expression level of all genes for that chromosome was calculated, separately, for the normal and abnormal samples. The fold change of abnormal to normal samples was then calculated, such that positive fold change values reflect an increase in expression in the aneuploid class and negative fold changes an increase in the relative expression of the normal class.

**Ontology Analysis**

Genes were compiled from the following four sources: Gene Ontology, Cancer Genome Project, PluriNet [[17](#_ENREF_17)], and Genomic Instability Set [[18](#_ENREF_18)]. The Gene Ontology database was queried for each ontology of interest by the GO ID using the GO ID/Term search. The entire list of gene ontologies including children consists of 280 unique ontologies, translated in R from the GO ID using the *go.db* library. From the Cancer Genome Project, all the genes of the “complete working list” from the cancer gene consensus were included. Genes that were listed with an associated cancer or syndrome were annotated under phenotype as cancer or cancer predisposition in our final gene list. Genes from the Genomic Instability set were listed as genomic instability and their associated function were included. Genes from the PluriNet were added and were annotated under phenotype as self-renewal. Ontologies were abbreviated and the phenotype translated to cancer, cancer disposition, self-renewal, and genomic instability. Significantly increased genes located on one of the trisomic chromosomes were then annotated with ontology and phenotype.

**Preparation of hPSCs for Karyotype Analysis**

Human PSC cultured in T-25 flasks were subjected to cytogenetic karyotype analysis. Cells were cultured to sub confluency with a growth medium change 24 hours before harvesting. 120 ng/mL of colcemid was added for 3 hours and 15 minutes at 37^o^C. Cells were rinsed with HBSS–(Ca/Mg) and dissociated with 0.05% trypsin. Dislodged cells were centrifuged at 1000rpm for 8 minutes. The cell pellet was gently resuspended in 0.075 M KCl solution and fixed in a solution containing 3:1 of methyl alcohol and glacial acetic acid at -20^o^C for 30 minutes followed by twice-repeated rinsing in fixative at 1000 rpm for 8 minutes. Cells were stored in fixative at -20^o^C until analysis. Metaphases were spread on microscope slides, and chromosomes were classified according to the International System for Human Cytogenetic Nomenclature (ISCN) using the standard G banding technique. For non-clonal analysis, >50 metaphases were examined. For clonal g-banding, 20 metaphases for each sample were examined.

**Clonality Assay**

For low-density survival assays, 1000 hPSC cells obtained after passing through a 40 μm filter were plated onto iMEFs in 35mm plates. To visualize hPSC colonies, cultures were stained for alkaline phosphatase on Day 7 after initial seeding. The number of colonies were counted using an inverted Nikon TS100 Microscope (Nikon, Melville, NY).

**Generation of ARHGDIA overexpression lines**

For lentiviral generation, Glycerol stock of Precision LentiORF ARHGDIA w/ Stop Codon (Open Biosystems) was stored at -80^o^C. 10 μl of ARHGDIA glycerol stock was used to inoculate 3-5 mls of LB Medium with ampicillin and agitated for 16 hours at 300 rpm at 37^o^C. 1 ml was further diluted in 250 ml, shaken at 300 rpm and incubated another 24 h at 37^o^C. The plasmid was extracted using the Qiagen Maxi Prep kit by following the manufacturer’s directions, and plasmid concentration quantitated using the BioMate3 UV-VIS Spectrophotometer (Thermo Scientific, Waltham, MA). Lentivirus was generated using HEK293 cells on a 10 cm cell culture plate. 80% confluent HEK293 cultures were transfected with 10 μg of the ARHGDIA plasmid, 7.5 μg of the packaging plasmid (psPAX2) (Addgene, MA), and 2.5 μg of the envelope plasmid (pMD.2) (Addgene, MA) using Roche transfection reagent, XtremeGENE 9. After 48 h exposure, the viral supernatant was collected, 0.45 μm filtered, and stored at 4^o^C for less than 24 hours. The viral supernatant was concentrated using the Lenti-X concentrator (Clontech, CA) by following the manufacturer’s protocol. The concentrated virus was resuspended in 500 μl of DMEM/F12 medium and aliquotted for storage at -80^o^C . One 50 μl aliquot was added to a 35mm petri dish containing hPSCs cultured on Matrigel™ or iMEFs along with the addition of 6 μg per ml of polybrene (Santa Cruz Biotechnology, CA). 24 hours later, virus containing medium was exchanged with the hPSC growth medium. Subsequently, the hPSCs monitored for GFP- positive expression by the Nikon Eclipse TE 2000-S inverted microscope (Nikon, Melville, NY). GFP- positive colonies were marked by objective- marker lens, manually selected, and propagated until use in experimentation.

**In vitro differentiation of hPSCs and histopathology of hPSC-derived embryoid bodies.**

To generate embryoid bodies (EBs), hPSCs were dissociated using 0.05% Trypsin and resuspended in growth medium without bFGF. EB formation was facilitated using suspension culture by a hanging drop method, where cells at a density of 5,000 cells per 20 μl drop were suspended from a Petri dish lid in 50-100 droplets with the addition of 10 μM ROCKi in the culture medium. After 3 days, the surviving EBs were transferred onto agar plates at in 20 ml of EB medium (hPSC growth medium without bFGF) to facilitate further differentiation with medium changes every 2 days for a total differentiation duration of 15-20 days. EBs were prepared for morphological analysis by fixation in 4.0% PFA in 1.5 mL microfuge tubes at 50-100 EBs per tube. EBs were rinsed with PBS to remove PFA, resuspended in 200 μL melted 4% low melting point agarose (Sigma Aldrich) at 42^o^C, and incubated for 2 h to allow settling. Final pelleting and agarose solidification were performed with brief room temperature centrifugation at 500g. Agarose embedded samples were removed as single plugs and processed by dehydration with increasing ethanol concentration to 100% followed by xylene and paraffination in a Leica TP1020 tissue processor. Hematoxylin and Eosin (H and E) staining was performed on microscope slide mounted 5 mm sections in a Leica Autostainer XL workstation (Leica Microsystems, Richmond, IL). Images were acquired using an Olympus BX51 microscope (Olympus, Center Valley, PA) using the default imaging parameters.

**Antibodies and Immunocytochemical Analysis**

HPSCs cultured on Matrigel™ or iMEFs in four chambered glass slides or cover slips in 35mm dishes were fixed via paraformaldehyde (PFA, 4%) in PBS. Permeabilization for intracellular markers was achieved with 0.2% Triton X-100 in PBS. 3% normal goat serum was used for blocking and to dilute antibodies. Fixed cells were incubated with primary antibodies: OCT4 (Santa Cruz Biotechnology, Santa Cruz, CA), SSEA4 (Millipore, Temecula, CA). Goat anti-mouse IgG, anti- rabbit conjugated to Alexa 488 or Alexa 594 (Molecular Probes, Eugene, OR) were used as secondary antibodies. Cells were counterstained with DAPI (4'-6-diamidino-2-phenylindole). Fluorescent images were acquired using a Cool- Snap EZ camera (Photometrics, Tucson, AZ) mounted on a Nikon Eclipse TE 2000-S inverted microscope (Nikon, Melville, NY) with NIS Elements BR software.

**RNA isolation, real time reverse transcription quantitative polymerase chain reaction and gene expression analysis**

RNA was isolated from hPSCs using DNA/RNA All Prep (Qiagen) and quantified using BioMate3 UV-VIS Spectrophotometer (Thermo Scientific, Waltham, MA) or Nanodrop 2000. cDNA was synthesized from 1 μg of RNA using cDNA High Capacity reverse transcription kit (Applied Biosystems, Foster City, CA). Transcriptional expression was assayed using Sybr Green quantitative real time RT-PCR (QPCR). QPCR was performed on a Bio-Rad CFX96 Touch Real-Time PCR Detection System. Comparative gene expression analysis (three replicates) was conducted using the ΔCT or ΔΔCT method depending on the analysis and samples. GAPDH was used for normalization in all analyses. Relative gene expression for hPSC experimental samples was assessed against hPSC controls and reported as fold change and the student’s T- test used for statistical significance testing.

**Alkaline Phosphatase Assay**

Staining for alkaline phosphatase was performed as per manufacturer instructions (Vector Laboratories, Burlingame, CA). Briefly, the hPSCs were rinsed with deionized water to remove traces of growth medium. The final solution was prepared by adding in order 2 drops of each constituent to 200mM Tris HCl buffer, pH 8.5. The hPSCs were incubated in the final mixture for 45 min in the dark and images were acquired using a Nikon DS-Fi1 camera mounted on a Nikon TS100 Microscope (Nikon, Melville, NY).

**Western Blot**

**At time of cell harvest and protein lysis, adherent cells were kept on ice and rinsed twice with ice-cold PBS+/+ (containing 0.9 mMCaCl2, 0.52 mMMgCl2 and 0.16 mM MgSO4) solution. After aspiration of the PBS+/+ solution, cells were lysed using the lysis buffer (Part# GL36) with protease inhibitor from the G-lisa kit, Cat# BK124 (Cytoskeleton Inc., CO). Upon addition of the lysis buffer, the cells were scraped, then spun down for 1.5 min at 11.6K rpm, and snap frozen in liquid nitrogen followed by stored at -80^o^C. 20 μl of the protein lysate was used to quantitate the protein concentration using the Precision Red Assay (Cytoskeleton, Inc.). For Western analysis, each lane on the gel was loaded with 30 μg of protein sample. Beta-tubulin anti-mouse (DSHB, Iowa) was used as the loading control and ARHGDIA anti-rabbit (Santa Cruz Biotechnology, Inc.) as the interrogating protein. Proteins were separated by SDS-PAGE and transferred onto nitrocellulose membranes (BioRad) for western blotting. The membranes were exposed to primary antibodies at 1:500 to 1:2,000 dilutions at 4^o^C overnight. Specific protein bands were detected and quantified using infrared-emitting conjugated secondary antibodies—anti-mouse 680 Alexa (Molecular Probes, Eugene, OR) or anti-rabbit IRDYE 800 (Rockland Immunochemicals, Gilbertsville, PA). Blots were exposed to secondary antibodies for 2 hours. Odyssey Infrared Imaging System and Image Studio Lite version 3.1 from Li-Cor Biosciences (Lincoln, NE) was used for image capture and densitometric analysis. For densitometric analysis, protein signal intensities were calculated and normalized against the loading control protein using the signal intensity of the lane divided by the maximum intensity of β- tubulin as the normalization ratio.**
