# Supplementary material for: ARHGDIA Confers Selective Advantage to Dissociated Human Pluripotent Stem Cells
Source: Stem Cells Dev. 2021 Jul 16;30(14):705–13. doi: 10.1089/scd.2021.0079 (PMC8309423; doi:10.1089/scd.2021.0079)
Supplement: Supplemental data [file Supp_Table1.docx]

SUPPLEMENTAL TEXT

**Table 1** Clonal genomic abnormalities during serial single-cell culture

| Cell Line | Passage # | Enzymatic # | Metaphase # | Karyotype (%) |
| --- | --- | --- | --- | --- |
| H1 | 68 | 29 | 20 | 46, XY |
| H1 | 97 | 58 | 20 | 46, XY,add(15)(p11.2) |
| H9 | 71 | 26 | 52 | 47, XX, +17 (67) |
| H9 | 150 | 105 | 20 | 48, XX,+12,+del(17)(p11.2) |
| BG01(v) | 69 | NR | 50 | 50, XXY (94); +3 (14); +11 (18); +12 (92); +14 (84); +17 (92); +20 (34); +der(16) (16) |
| iPSC | 45 | 31 | 20 | 48, XXY,+17 |

HPSCs dissociated as single cells and cultured on iMEFs. The H1 and H9 lines were continuously passaged and karyotyped at two different passage points. H1 at passage 97 gained a segmental duplication of 15p11.2. H9 at early enzymatic passage, E-26, was 67 percent mosaic for trisomy 17 and subsequently gained an additional chromosome 12. BG01(v) exhibits hyper-diploidy and culture mosaicism. The iPSC gained an X and chromosome 17. Non-clonal analysis was performed on BG01(v) and H9 passage 71 lines, with 50 and 52 metaphase spreads analyzed, respectively. The (%) is the percentage of cells in culture expressing the indicated genomic alteration. Abbreviations: add- addition, del- deletion, der- derivative, E- enzymatic passage, NR- not recorded.
