# Supplementary material for: ARHGDIA Confers Selective Advantage to Dissociated Human Pluripotent Stem Cells
Source: Stem Cells Dev. 2021 Jul 16;30(14):705–13. doi: 10.1089/scd.2021.0079 (PMC8309423; doi:10.1089/scd.2021.0079)
Supplement: Supplemental data [file Supp_Fig1.docx]

**Figure 1. Trisomy enrichment of significantly increased genes.** In differential gene expression analysis between the euploid H1 line and aneuploid BG01(v) line, 450 genes were found to be increased (black) and 393 decreased (white) between the cell lines. We normalized each chromosome’s count by dividing by the number of array genes for the respective chromosome. Chromosomes 12, 14, 17, and X show a marked increase in the normalized ratio of genes that are differentially regulated and have an increased fold change. For normalized ratio between disomic and trisomic chromosomes, p-value = .0046.
