# Supplementary material for: ARHGDIA Confers Selective Advantage to Dissociated Human Pluripotent Stem Cells
Source: Stem Cells Dev. 2021 Jul 16;30(14):705–13. doi: 10.1089/scd.2021.0079 (PMC8309423; doi:10.1089/scd.2021.0079)
Supplement: Supplemental data [file Supp_Fig2.docx]

**
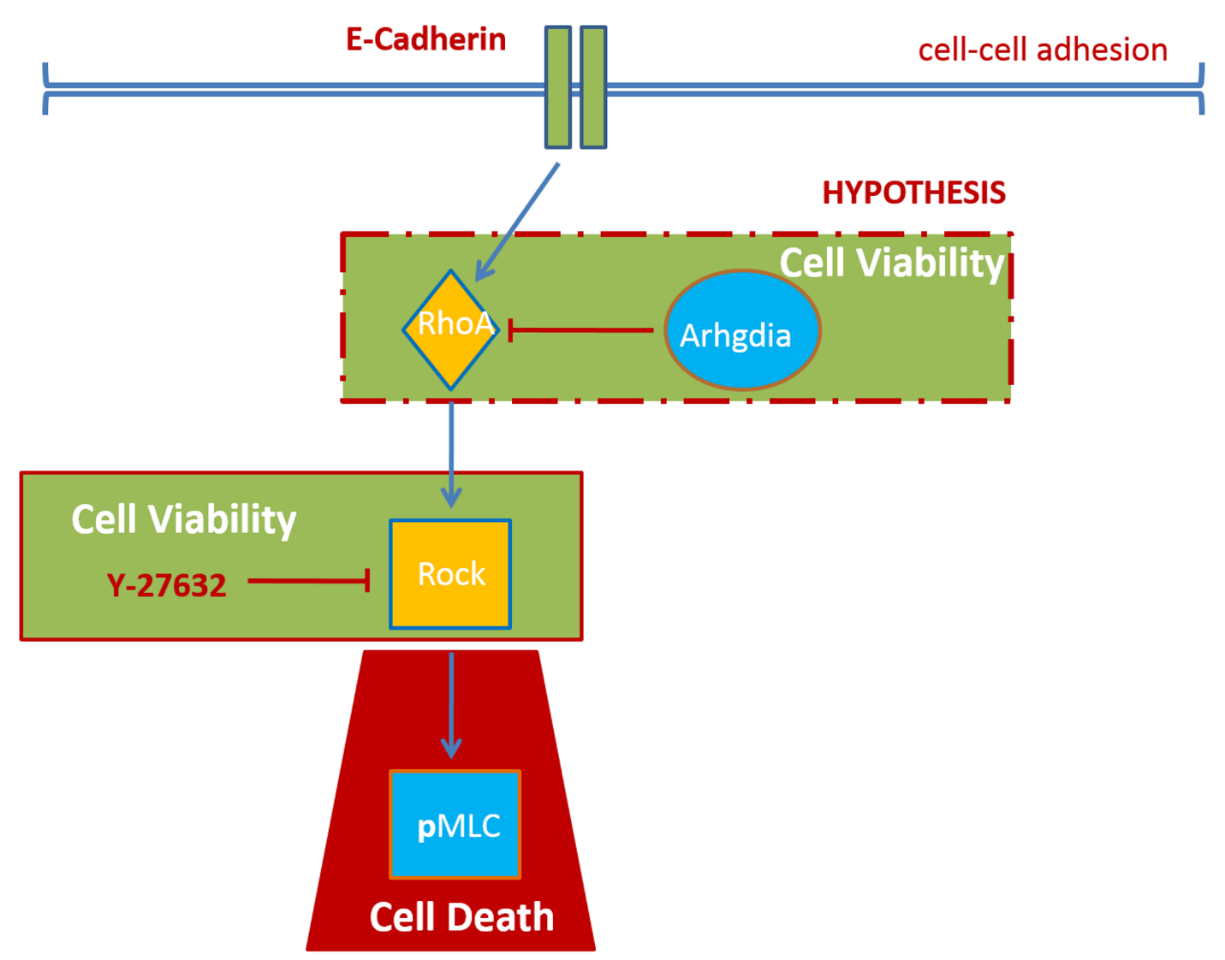
**

**Figure 2. ARHGDIA inhibition of RHO signaling.** Loss of cell-cell contact during single cell dissociation leads to cell death through activation of Rho signaling. Phosphorylation of myosin light chain by ROCK induces cytoplasmic membrane blebbing and rupture. ARHGDIA is a potent inhibitor of RHOA activation. ARHGDIA maintains the inactive RHOA-GDP bound state by preventing the switch to active RHOA-GTP. We hypothesize that overexpressing ARHGDIA will reduce RHOA activation, and therefore reduce ROCK associated, pMLC- dependent, apoptosis leading to increased viability of hPSCs. Abbreviations: pMLC- phosphorylated myosin light chain, ROCK- rho-associated coil coiled protein kinase.
