# Supplementary material for: ARHGDIA Confers Selective Advantage to Dissociated Human Pluripotent Stem Cells
Source: Stem Cells Dev. 2021 Jul 16;30(14):705–13. doi: 10.1089/scd.2021.0079 (PMC8309423; doi:10.1089/scd.2021.0079)
Supplement: Supplemental data [file Supp_Fig3.docx]

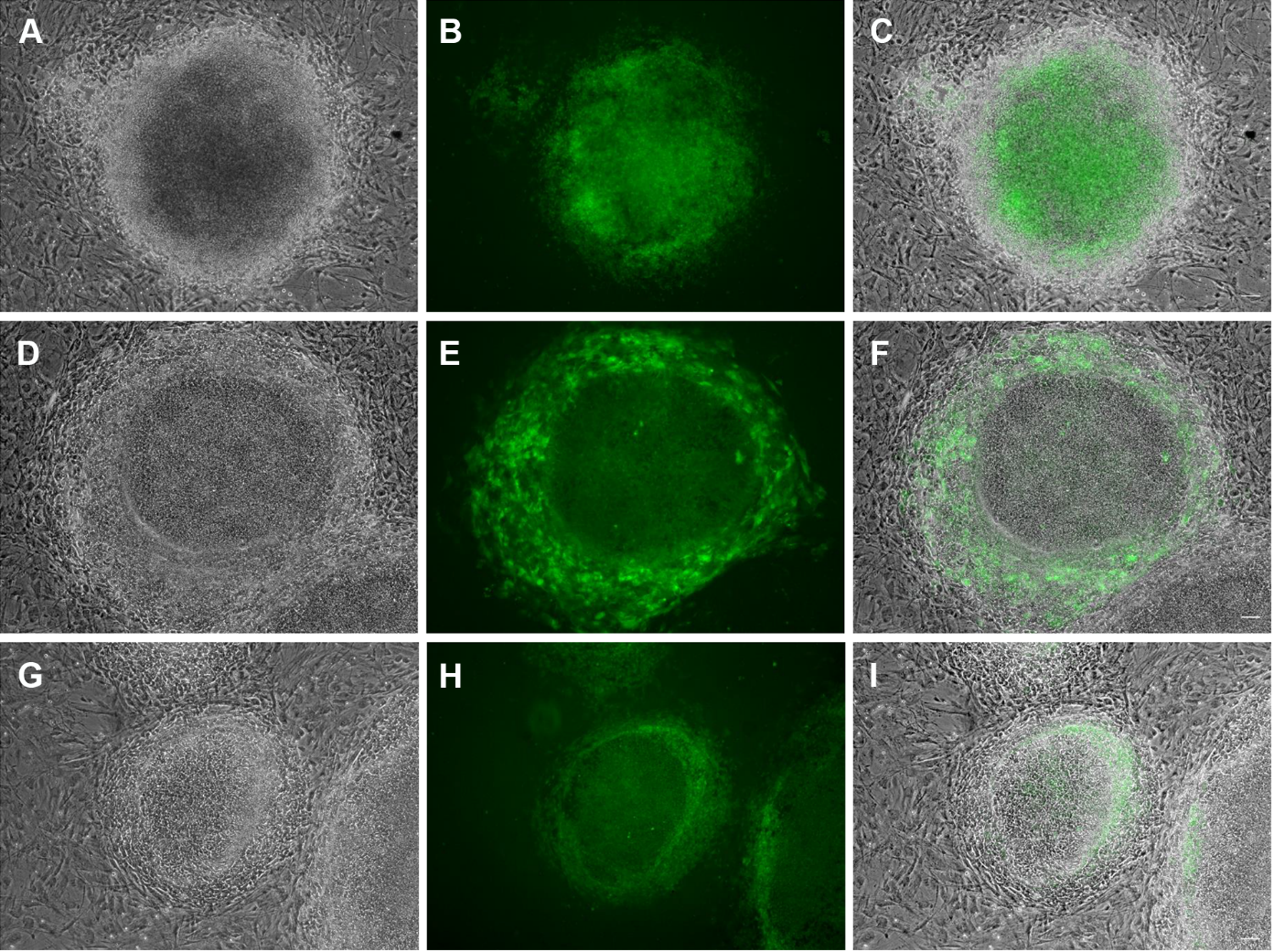


**Figure 3. H9 (Arg) colonies exhibits increased cell- cell contact and mounded morphology.** Each line exhibits standard pluripotent stem cell colony formation. H9 (Arg) (A-C) has marked increased multi- cell layer morphology as demonstrated by the increased density in phase contrast image (A) and fluorescent image (B) and overlay (C). H9 (GFP) (D-F) exhibits standard monolayer colony morphology concave to surrounding iMEF layer. BG01 (Arg) (G-I) exhibits standard monolayer colony morphology concave to surrounding iMEF layer. Scale bar= 100μm.

**β-tub- β-tubulin, WT- wild type, v- genomic variant, GFP- green fluorescent protein.**
