# Supplementary material for: ARHGDIA Confers Selective Advantage to Dissociated Human Pluripotent Stem Cells
Source: Stem Cells Dev. 2021 Jul 16;30(14):705–13. doi: 10.1089/scd.2021.0079 (PMC8309423; doi:10.1089/scd.2021.0079)
Supplement: Supplemental data [file Supp_Fig9.docx]

**Figure 9. ARHGDIA protein expression in transduced and variant hPSC lines. Western blot analysis on ARHGDIA protein expression in H9s and BG01s (A). The numbered lanes of the western blot (A) correspond to the numbered rows in the densitometric analysis (B). Odyssey Infrared Imaging System and Image Studio Lite version 3.1 from Li-Cor Biosciences (Lincoln, NE) was used for image capture and densitometric analysis. For densitometric analysis, the signal intensities were normalized against maximum β-tubulin expression. Fold change values for H9 (Arg) and BG01 (Arg) are relative to H9 (GFP) and BG01 (GFP) and fold change values for H9(v) and BG01(v) are relative to H9 (WT) and BG01 (WT). β -tubulin= 55 kDa and ARHGDIA= 26 kDa. Abbreviations: Arg- ARHGDIA, β-tub- β-tubulin, WT- wild type, v- genomic variant, GFP- green fluorescent protein.**
