# Supplementary material for: ARHGDIA Confers Selective Advantage to Dissociated Human Pluripotent Stem Cells
Source: Stem Cells Dev. 2021 Jul 16;30(14):705–13. doi: 10.1089/scd.2021.0079 (PMC8309423; doi:10.1089/scd.2021.0079)
Supplement: Supplemental data [file Supp_Fig4.docx]

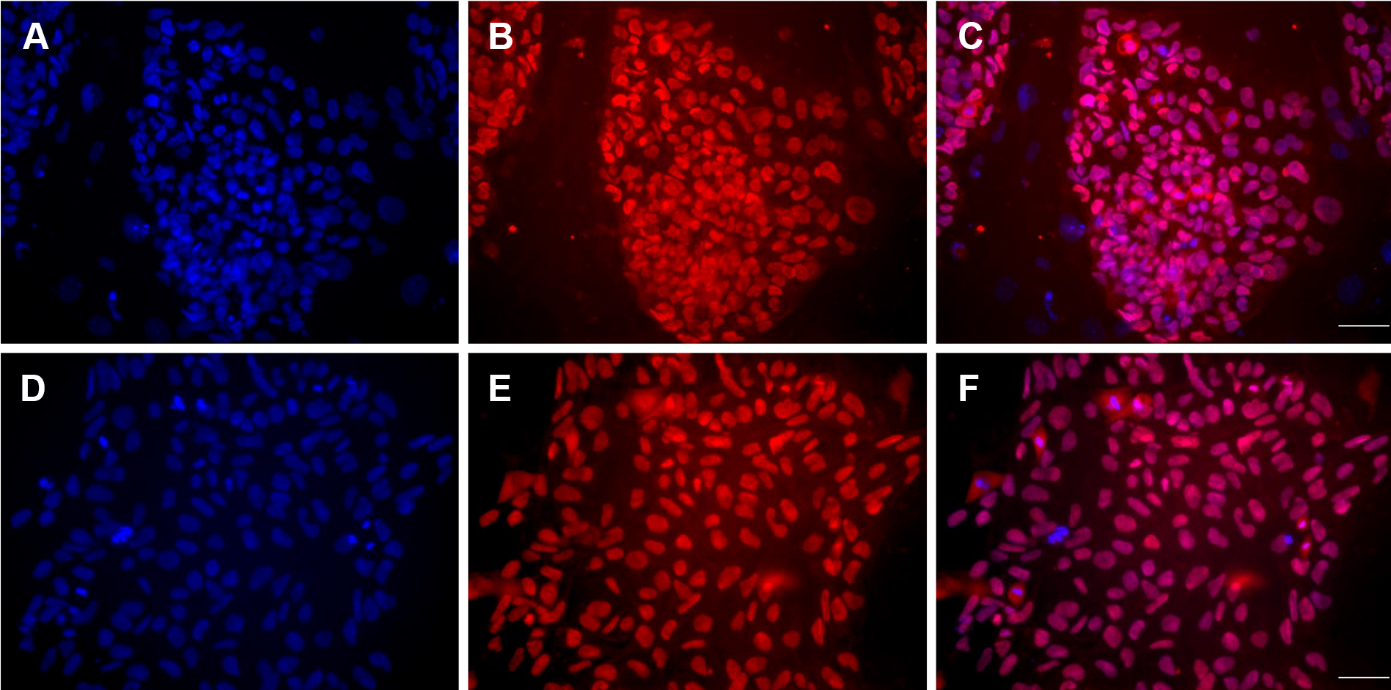


**Figure 4. hPSC (Arg) lines self- renew and express OCT4.** Positive nuclear expression of the OCT4 transcription factor in H9 (Arg) lines (A-C) and BG01 (Arg) lines (D-F). H9 (Arg) and BG01 (Arg) lines were propagated greater than 20 and 10 passages, respectively. DAPI- blue (A, D), OCT4- red (B, E), and DAPI/OCT4 nucleus overlay (C, F). Scale bar= 50 μm.

**β-tub- β-tubulin, WT- wild type, v- genomic variant, GFP- green fluorescent protein.**
