# Supplementary material for: ARHGDIA Confers Selective Advantage to Dissociated Human Pluripotent Stem Cells
Source: Stem Cells Dev. 2021 Jul 16;30(14):705–13. doi: 10.1089/scd.2021.0079 (PMC8309423; doi:10.1089/scd.2021.0079)
Supplement: Supplemental data [file Supp_Fig5.docx]

**
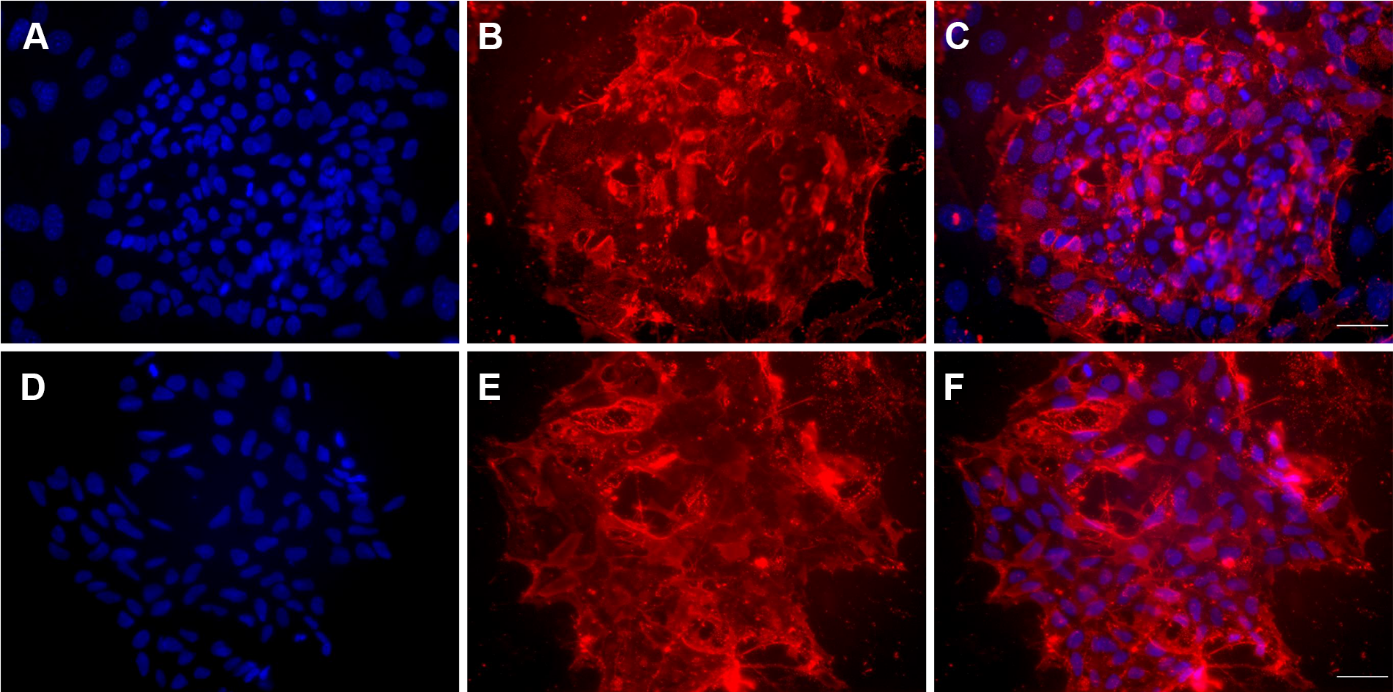
**

**Figure 5. hPSC (Arg) lines self- renew and express SSEA4.** Positive expression of the cell surface marker, SSEA4, in H9-arg+ lines (A-C) and BG01-arg+ lines (D-F). The H9 (Arg) and BG01 (Arg) lines were propagated greater than 20 and 10 passages, respectively. DAPI- blue (A, D), SSEA4- red (B, E), and DAPI/SSEA4 overlay (C, F). Scale bar= 50 μm.

**β-tub- β-tubulin, WT- wild type, v- genomic variant, GFP- green fluorescent protein.**
