# Supplementary material for: ARHGDIA Confers Selective Advantage to Dissociated Human Pluripotent Stem Cells
Source: Stem Cells Dev. 2021 Jul 16;30(14):705–13. doi: 10.1089/scd.2021.0079 (PMC8309423; doi:10.1089/scd.2021.0079)
Supplement: Supplemental data [file Supp_Fig6.docx]

**Figure 6. H9 (Arg) s.1 demonstrates competitive advantage under single- cell passaging.**

H9 (Arg) lines was maintained on iMEFs under manual passage (E-0) then switched to single- cell dissociation (E-1 to E-6). By flow cytometry, the percentage of GFP positive cells was measured at each passage to assess the proportion of cells overexpressing ARHGDIA. The distribution of GFP signal intensity is bimodal with GFP- high and GFP- low fractions. The H9 (Arg) GFP- high exhibits increased competitive advantage relative to H9 (Arg) GFP- low.

**β-tub- β-tubulin, WT- wild type, v- genomic variant, GFP- green fluorescent protein.**
