# Supplementary material for: ARHGDIA Confers Selective Advantage to Dissociated Human Pluripotent Stem Cells
Source: Stem Cells Dev. 2021 Jul 16;30(14):705–13. doi: 10.1089/scd.2021.0079 (PMC8309423; doi:10.1089/scd.2021.0079)
Supplement: Supplemental data [file Supp_Fig8.docx]

**Figure 8. ARHGDIA is overexpressed in BG01 (Arg) and H9 (Arg) lines. Using real-time PCR, the BG01 (Arg) and H9 (Arg) lines overexpress ARHGDIA transcripts relative to their respective set of controls, BG01 (GFP), BG01 (WT), H9 (GFP), and H9 (WT). For each cell line n= 3.**
